# Kinetics of T Helper Subsets and Associated Cytokines Correlate Well with the Clinical Activity of Graft-Versus-Host Disease

**DOI:** 10.1371/journal.pone.0044416

**Published:** 2012-09-05

**Authors:** Su-Peng Yeh, Yu-Min Liao, Wen-Jyi Lo, Chiao-Lin Lin, Li-Yuan Bai, Chen-Yuan Lin, Ching-Yun Hsieh, Yu-Chien Chang, Yu-Ting Huang, Chang-Fang Chiu

**Affiliations:** 1 Stem Cell Research Lab, Department of Medical Research, China Medical University Hospital, Taichung, Taiwan; 2 Division of Hematology and Oncology, Department of Internal Medicine, China Medical University Hospital, Taichung, Taiwan; 3 China Medical University, Taichung, Taiwan; University of California Los Angeles, United States of America

## Abstract

**Background:**

CD4^+^interferon (IFN)-γ^+^ T cell (Th1) and CD4^+^interleukin (IL)-4^+^ T cell (Th2) polarizations are crucial in the pathogenesis of graft-versus-host disease (GVHD). However, this hypothesis is largely based on animal experiments of Parent-into-F1 GVHD model. The causal relationship between kinetics of Th1, Th2 and associated cytokines and the clinical activity of GVHD in a real world situation remains unknown.

**Methodology:**

Peripheral blood was collected every week prospectively from Day 0 to Day 210 (patients without GVHD) or Day 300 (patients with chronic GVHD) after allogeneic peripheral blood stem cell transplantation in consecutive 27 patients. The frequencies of Th1 and Th2 within CD4^+^ T cells were determined by flow cytometry and pplasma IFN-γ, IL-12, IL-4, and IL-10 were determined by ELISA.

**Principal Findings:**

Kinetics of Th1, Th2 frequency, and the plasma IL-10 and IFN-γ more commonly coincided with, rather than predicted, the activity of GVHD. These markers are significantly higher when acute or chronic GVHD developed. The kinetics of IL-10 is especially correlated well with the activity of GVHD during clinical course of immunosuppressive treatment. For patients with hepatic GVHD, there is a positive correlation between plasma IL-10 levels and the severity of hepatic injury. The frequency of Th2 is also significant higher in acute GVHD and tends to be higher in chronic GVHD. Interestingly, there is a very good positive correlation between the frequency of Th1 and Th2 (r = 0.951, p<0.001). The plasma level of IL-4 and IL-12 are not associated with the activity of GVHD.

**Conclusions:**

The frequency of Th1, Th2 within CD4^+^ T cells and plasma IL-10 and IFN-γ are good biomarkers of GVHD. Plasma IL-10 can also be used to monitor the therapeutic responsiveness. Furthermore, both Th1 and Th2 likely contribute to the pathogenesis of GVHD.

## Introduction

GVHD is a major complication after allogeneic hematopoietic stem cell transplantation (HSCT). It is a dynamic course of tissue injuries and many organs can be involved simultaneously or sequentially. Tissue biopsy is therefore needed to confirm the diagnosis and determine the severity of GVHD. However, repetitive tissue biopsies to evaluate the activity of GVHD are unpractical during the protracted course of GVHD. Clinical manifestations, although frequently non-specific and can be confused with other medical problems, remain the major determinants in assessing the status of GVHD. A reliable biomarker, which is readily available without using invasive procedures, can facilitate not only the management but also the understanding of GVHD.

Historically convincing evidence indicates that T-cells contained in donor graft or subsequently derived from stem cells initiate the GVH reaction [1], [2]. More recently, T-cell activation in patients with acute GVHD have a CD4 subset imbalance favoring T helper1 (Th1) cells, which secrete type 1 cytokines interleukin (IL)-2, IL-12, interferon (IFN)-γ, and tumor necrosis factor-α [3]–[5]. On the other hand, the activation of T helper2 (Th2), which secrete type 2 cytokines IL-4 and IL-10, with subsequent Th2 humoral immune response may be responsible for the development of chronic GVHD [6], [7]. Both Th1 and Th2 are derived from naïve T cells and the most clearly defined differentiation inducers are themselves cytokines: IFN-γ and IL-12 for Th1, and IL-4 and IL-10 for Th2 [8], [9]. Elevated plasma IL-10 [10]–[16] and IFN-γ [13], [15], [17] had been found clinically in patients having GVHD. It is therefore reasonable to hypothesize that serial post-transplant Th1, Th2 subsets and associated cytokines (including IFN-γ, IL-12, IL-4 and IL-10) monitoring may elucidate the immunological network of GVHD and thus improve our management of GVHD. Nevertheless, this hypothesis was largely based on animal experiments of two Parent F1 GVHD model rather than real world situation. Furthermore, most of the published studies [10]–[19] examining post-transplant cytokines had some weak points which might make interpretation and clinical application difficult. For example, many of these studies examined the immune status at a single or only few time points following transplant [10], [15], [16], [18], [19], which is difficult to reflect the whole picture of the post-transplant course. Also, the timing sequence between changing immune status and clinical activity of GVHD was not well-illustrated in all these studies and so the fundamental question remains unanswered whether high concentrations of cytokines in the blood are a cause or a consequence of GVHD. It was also unknown whether level of cytokines is associated with severity of GVHD [10], [12]–[17], [19]. Infection is frequently seen in transplant recipients and levels of IL-10 [20] and IFN-γ [18] may be increased during infectious episodes irrespective of GVHD. In our preliminary study, increased IL-10 levels were also found in two patients having bacteremia but no GVHD (Figure S1A and S1B). However, many of these studies [11], [12], [14], [17], [18] enrolled patients with infection or fever. Finally, most of these studies did not examine cytokines in patients with chronic GVHD [10]–[16], [18], [19] and none of these studies examined Th1 and Th2 levels simultaneously, although the pathogenetic role of Th1 and Th2 were frequently speculated [10]–[14], [16], [18]. In the current study, we collected patients’ peripheral blood weekly from Day 0 to Day 210 (patients without GVHD) or Day 300 (patients with chronic GVHD) after allogeneic transplantation. Patients getting infection after myeloid engraftment were excluded from analysis. With these efforts, we can continuously monitor the dynamic change of immune status (not only plasma IFN-γ, IL-4, Il-10, IL-12 levels, but also the Th1, and Th2 frequencies within total CD4^+^ T cells simultaneously) and then to establish the relationship between kinetics of immune status and the clinical activity of both acute and chronic GVHD.

## Design and Methods

### Ethics Statement

This study was conducted under the condition approved by local institutional review board (IRB) of China Medical University Hospital.

### Patients

Consecutive 27 adults receiving allogeneic HSCT between Feb. 2004 and Dec. 2006 at China Medical University Hospital were enrolled in this study. Peripheral blood was collected from each patient weekly on a calendar basis from Day 0 to Day 210 (if no GVHD) or Day 300 (if chronic GVHD developed). All the 27 patients received peripheral blood stem cell transplantation from HLA-matched siblings and GVHD prophylaxis consisted of standard cyclosporine-A and short-course methotrexate. The chimerism status of recipients was determined by cytogenetic study for sex-mismatched transplant and short tandem repeat polymorphism for sex-matched transplant. 6 patients were excluded from this study due to death before myeloid engraftment (1), primary graft failure (1), early relapse of disease (2), and grade 3 hemorrhagic cystitis on hyperbaric oxygen therapy (2). Since our preliminary data showed the plasma IL-10 levels may be increased during blood-stream infection irrespective of GVHD (Figure S1A and S1B) [21], another 5 patients with severe infection such as blood-stream infection or image-documented pneumonia were also excluded from this study. The remaining 16 patients who had uneventful post-transplant course or “pure” GVHD were selected for further investigation. They were 4 patients of no GVHD, 3 patients having acute GVHD, 4 patients having both acute and chronic GVHD, and 5 patients having de novo chronic GVHD. The characteristics (including the onset day, grade, and target organs of GVHD) of these 16 patients were showed in supporting table (Table S1). The diagnosis of GVHD was made based on the histological and clinical findings. For patients with liver being the major target of GVHD (including patient No. 10, 11, 12, 15, and 16), liver biopsy was done to confirm the diagnosis of hepatic GVHD. Grading of acute and chronic GVHD was defined according to the criteria described by Glucksberg [22] and Shulman et al [23] respectively. The initial treatment for both acute and chronic GVHD is prednisolone 0.5 to 1 mg/kg/day.

### Measurement of Plasma IFN-γ, IL-4; IL-10, and IL-12 Concentration

Peripheral blood obtained from patients were centrifuged immediately and the plasma specimens were then stored at −80°C. At examination, the plasma was thawed at 37°C and the plasma level of IFN-γ, IL-4; IL-10, and IL-12 were determined by high sensitivity enzyme-linked immunosorbent assay (ELISA, R&D, Minneapolis, MN, US). For the 4 patients of no GVHD (patient No. 3, 4, 5, and 6), the criteria for choosing the time points for cytokine analysis were taken on a calendar basis, i.e., every 2 weeks from Day 0 to Day 210. A total of 64 plasma specimens were thawed for analysis and this data would be served as “No GVHD-external control”. For the other 12 patients of acute and/or chronic GVHD, the criteria for choosing the time points for cytokine analysis were taken on an event-driven basis, i.e., the week immediately before the clinical onset of GVHD, every 1–2 weeks during episodes of GVHD, and every 2–4 weeks when GVHD was inactive (either before the onset or after the resolution of GVHD). A total of 327 plasma specimens from these 12 patients were thawed for analysis. Matching the time points of blood withdrawing to patients’ clinical background, 192 samples were collected at a clinical condition of no GVHD (the data would be served as “No GVHD-internal control”), 49 at acute GVHD, and 86 at chronic GVHD. According to the manufacturer, the reliable detecting limit of IL-4; IL-10, and IL-12 were 0.13 pg/ml, 0.5 pg/ml, and 0.5 pg/ml respectively. The reliable detecting limit for IFN-γ was 3.1 pg/ml for the first 264 samples and 2.7 pg/ml for the last 127 samples due to the use of different batch of ELISA kit.

### Measurement of Frequency of Th1 and Th2 Subsets in CD4^+^ T Cells

The Th1 and Th2 were determined by intracellular cytokine staining technique followed by flow cytometric analysis as reported previously [24]. In brief, the mononuclear cells of peripheral blood were obtained by centrifugation on a Ficoll-Paque gradient (Pharmacia; Peapack, NJ, USA) and then cryopreserved in liquid nitrogen. At examination, the buffy coat was thawed at 37°C and Brefeldin A (BFA) was added to block intracellular transport. The cells were incubated in RPMI1640 medium for 4 hours with or without adding activating agent phorbol 12-myristate 13-acetate [PMA] + ionomycin [I]. Cells were then stained with surface antigens (CD4, BD, Biosciences)-specific monoclonal antibodies. After adding FACS Lysing solution to lyse red cells, the cells were permeabilized and fixed with Cytofix/Cytoperm kit (BD Pharmingen). Fluorescence-conjugated anti-IFN-γ and IL-4 monoclonal antibodies were then added and the samples were analyzed on a FACS Calibur (BD Biosciences). CD4^+^IFN-γ^+^ cells and CD4^+^IL-4^+^ cells were designated as Th1 and Th2 respectively. The Th cells with or without in vitro PMA+I stimulation were designated as stimulated Th (sTh) and unstimulated Th (uTh) cells respectively. Quadrant markers were set based on background staining of matched control antibodies (BD Biosciences). A total 59 blood specimens (from 3 patient of no GVHD and 7 patients with GVHD) were selected for examination. For the 3 patients (case No. 3, 5, 6) of no GVHD, the criteria for choosing the time points of T cell subsets analysis were taken on a calendar-basis (week 4, 8, 16, 24, and 28). A total of 15 samples were examined and the data would be served as “No GVHD-external control”. For the other 7 patients with GVHD (case No. 8, 9, 10, 11, 12, 15 and 16), the criteria for choosing the time points for T-cell subset analysis were taken on an event-driven basis. We selected the following time points for analysis: baseline (usually 1–2 weeks prior to the clinical onset of GVHD), clinical onset and peak of GVHD, and resolution of clinical GVHD. A total of 44 samples were examined, including 15 collected at a clinical condition of no GVHD (and the data would be served as “No GVHD-internal control”), 15 at acute GVHD, and 14 at chronic GVHD.

### Statistics

The significance of difference between plasma IL-10, the frequency of Th1, Th2, Th1/Th2 ratio and status of GVHD (“No GVHD-external control”, “No GVHD-internal control”, acute GVHD, and chronic GVHD) was determined by student *t*-test. To examine the relationship between plasma IL-10 level and the severity of hepatic injury in the 6 patients with GVHD involving liver (patient No. 9, 10, 11, 12, 15, and 16), the significance of difference between plasma IL-10 level and no to mild hepatic injury (defined as total bilirubin <34.2 µmol/L and ALT <60 U/L), moderate hepatic injury (defined as total bilirubin between 34.2 and 102.6 µmol/L and/or ALT between 60 and 160 U/L), and severe hepatic injury (defined as total bilirubin >102.6 µmol/L and/or ALT >160 U/L) was determined by student *t*-test. The significance of difference between incidence of detectable plasma IFN-γ and status of GVHD was determined by Chi-Square test. To examine the relationship between plasma IL-10 and IFN-γ level at the same time point, the significance of difference between plasma IL-10 level and each category of plasma IFN-γ (undetectable, detectable but lower than 6 pg/ml, ≥ 6 pg/ml) was determined by student *t*-test. The correlation between the frequency of Th1 and Th2 was determined by Pearson correlation analysis using SPSS software.

## Results

### Interleukin 10

**Figure 1:**
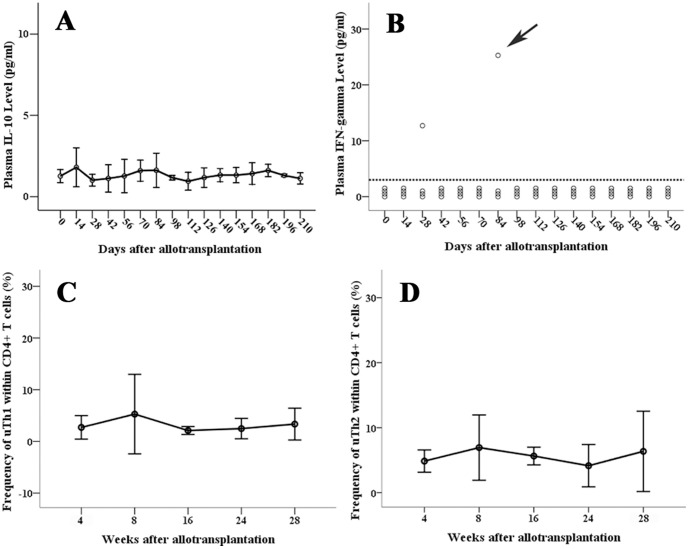
Immunological parameters of patients having no GVHD (No GVHD- external control). Serial plasma IL-10 (1A), IFN-γ (1B), and the frequency of uTh1 (1C) and uTh2 (1D) within CD4^+^ T cells during the post-transplant course of patients having no GVHD. Error bars in figure 1A, 1C, and 1D represent 2X the standard error of the mean. The dash line on figure 1B represents the detected limit of plasma IFN-γ. Arrow on figure 1B represents the concomitant herpes zoster infection of patient No. 4 at that time point.

**Figure 2:**
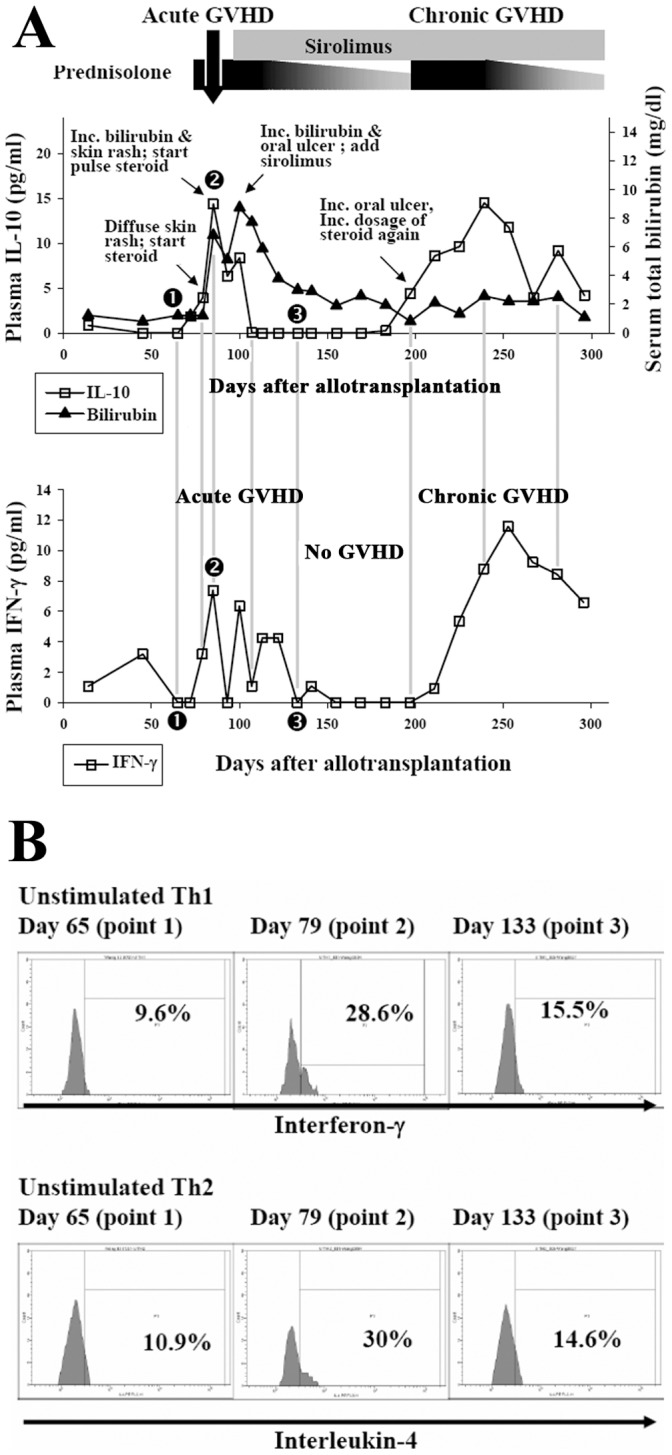
A patient with acute and chronic GVHD (Patient No. 10). Good correlation between plasma IL-10, IFN-γ levels (figure 2A), the frequency of uTh1, uTh2 (figure 2B) and clinical course of a patient having acute and chronic GVHD. Big arrow on the top of Figure 2A indicates pulse prednisolone therapy. Label 1, 2, and 3 on figure 2A (as well as in other figures) indicate the time points for which the frequencies of uTh1 and uTh2 within total CD4^+^ T cell population were examined and the results were showed as histogram in figure 2B.

**Figure 3:**
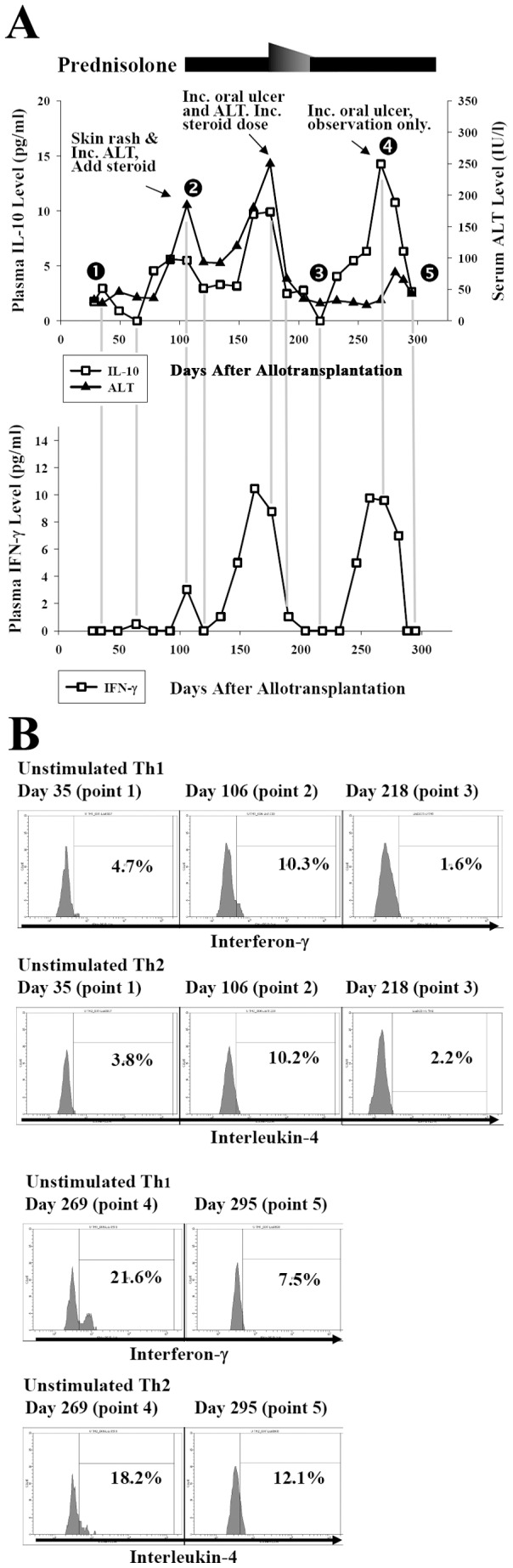
A patient with recurrent flare-up of chronic GVHD (Patient No. 11). Plasma IL-10 and IFN-γ levels correlate well with the clinical course of GVHD. Besides, the frequencies of uTh1 and uTh2 increase when patient had active GVHD (Points 2 and 4) and decrease when GVHD improved (Points 3 and 5). ALT: alanine aminotransferase.

**Figure 4:**
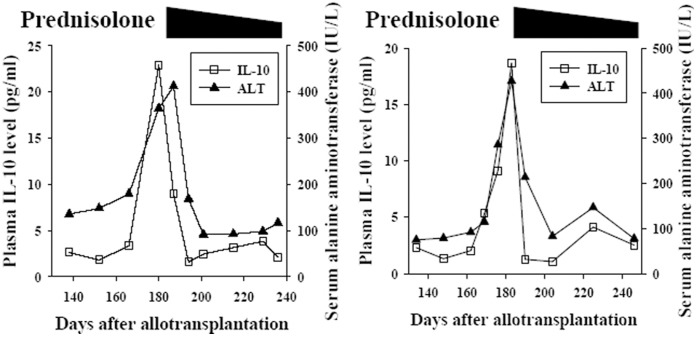
Prompt response of plasma IL-10 to effective immunosuppressive therapy in 2 patients with hepatic GVHD (4A: Patient No. 15; 4B: Patient No. 16). Plasma IL-10 increases in two patients with pathologically documented hepatic GVHD. After prednisolone treatment, the decrease of plasma IL-10 is prompt and earlier than clinical improvement of hepatic function.

**Figure 5:**
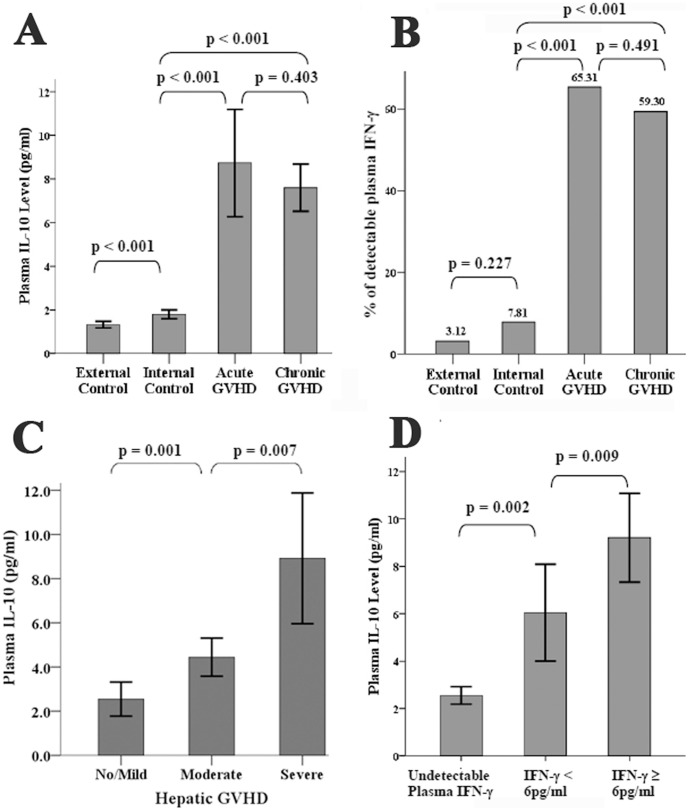
Correlation between plasma IL-10, IFN-γ and GVHD. GVHD is associated with significant higher level of plasma IL-10 (figure 5A, student *t*-test) and detectable plasma IFN-γ (figure 5B, Chi-Square test). Plasma IL-10 levels are also higher in patients with more severe hepatic GVHD (Figure 5C, student *t*-test). At the same time point, higher plasma IFN-γ is associated with significantly higher plasma IL-10 (figure 5D, student *t*-test). Bar represents mean and error bar represents 2X standard error of the mean (figure 5A/5C/5D).

Plasma IL-10 level correlates well with the clinical activity of GVHD. Of the 4 patients without GVHD, the plasma IL-10 levels were very low and below 4 pg/ml before Day 210. Figure 1A shows the mean IL-10 (±2S.E.) at each time point of these patients. The serial post-transplant IL-10 level of individual patient without GVHD (patient No. 3) is shown in supporting figure S2A as example. Of patients who had acute and/or chronic GVHD, plasma IL-10 level increased dramatically during the period of GVHD and decreased when GVHD improved (figure 2A and 3A). For patients of GVHD involving liver and/or oral cavity and had good response to corticosteroid treatment, the decrease of plasma IL-10 was prompt and was earlier than clinical improvement of hepatic function and oral mucositis (acute GVHD of figure 2A, and figure 4 as examples). For patients’ GVHD responded less well to immunosuppressive therapies, clinical symptoms of GVHD persisted along with persistently elevated plasma IL-10 levels (chronic GVHD of figure 2A as example: persistently high plasma IL-10 level between Day 200 and Day 250). Of all blood specimens (n = 391) examined, the plasma IL-10 was significantly higher when patients had acute or chronic GVHD (p<0.001). However, plasma IL-10 was not significantly different between acute and chronic GVHD (p = 0.403, figure 5A). Interestingly, mean IL-10 level (mean ± standard error) of “No GVHD-internal control” was also significantly higher than that of “No GVHD-external control” (1.798±0.103 vs 1.317±0.076, p<0.001). For patients with hepatic GVHD, the plasma IL-10 levels were significantly higher when patient had more severe hepatic injury. The plasma IL-10 level (mean ± standard error) during periods of no to mild, moderate, and severe hepatic GVHD were 2.54±0.39, 4.44±0.43, and 8.92±1.48 pg/ml respectively (p<0.05, figure 5B). Using 4 pg/ml as cut off value, a plasma IL-10 level lower than 4 pg/ml is associated with a 95% negative predict rate of GVHD. A plasma IL-10 level higher than 4 pg/ml is associated with an 88.5% positive predict rate and 76.9% detected sensitivity of GVHD.

### Interferon-γ

Plasma IFN-γ level also correlates well with the clinical activity of GVHD in most of the patients. Of the 64 specimens collected from the 4 patients without GVHD, detectable IFN-γ was found in only 2 of them (3.1%, figure 1B). Tracing back the clinical history, one point of “high IFN-γ” coincided with an acute episode of herpes zoster infection. Only one point of “high IFN-γ” was unexplained. On the other hand, plasma IFN-γ increases when acute or chronic GVHD developed (figure 2A and 3A). Of the other 327 specimens collected from 12 patients with GVHD, there were 192 “No GVHD-internal control”, 49 acute GVHD, and 86 chronic GVHD and plasma IFN-γ was detected in 7.8%, 65.3%, and 59.3% of them respectively. The detectable rate was significantly higher when GVHD developed (p<0.001, figure 5C). An undetectable plasma IFN-γ is associated with a 91.6% negative predict rate of GVHD. A detectable plasma IFN-γ level is associated with a 78% positive predict rate and 60% detected sensitivity of GVHD. The dynamic changes of plasma IFN-γ levels almost followed the same pace with IL-10, i.e., the plasma IFN-γ and IL-10 going up or going down at the same time in most occasions (figure 2A and 3A). Of the same blood specimen, a higher plasma IFN-γ level is associated with a significantly higher plasma IL-10 level (figure 5D).

### Interleukin-4 and Interleukin-12

Almost all the plasma IL-4 and IL-12 levels examined were below the detectable limit irrespective the clinical activity of GVHD (figure S3).

### T Helper 1 and T helper 2 Subsets

**Figure 6:**
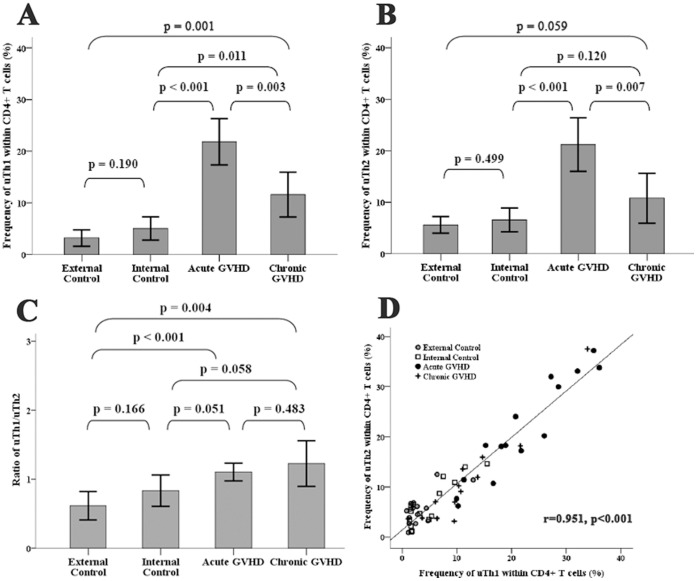
Correlation between uTh1, uTh2, and GVHD. The frequencies of uTh1 and uTh2 are significantly higher when patients had GVHD (figure 6A/6B, student *t*-test). The ratio of uTh1/uTh2 is significant lower in “No GVHD-external control” and tends to be lower in “No GVHD-internal control” when comparing to that of acute and chronic GVHD (figure 6C, student *t*-test). Pearson correlation test shows perfect positive correlation between the frequency of uTh1 and uTh2 (figure 6D). Bar represents mean and error bar represents 2X standard error of the mean. (figure 6A/6B/6C).

The dynamic changes of frequency of Th1 and Th2 within CD4^+^ T cell also correlated well with the clinical activity of GVHD. For patients without GVHD (both “No GVHD-external control” and “No GVHD-internal control”), the uTh1 and uTh2 were detected only in small numbers and the frequencies of uTh1 and uTh2 within total CD4^+^ cells were lower than 10% in most occasions (figure 1C, 1D, point 1 of figure 2B and 3B, and figure S2B). By contrary, both uTh1 and uTh2 cells can be easily detected in the blood when GVHD developed (point 2 of figure 2B, point 2 and 4 of figure 3B). If GVHD responded well to the immunosuppressive therapy, the frequency of uTh1 and uTh2 decreased rapidly (point 3 of figure 2B, point 3 and 5 of figure 3B). Taking together, the frequencies of uTh1 within total CD4^+^ T cells were significantly higher when acute and chronic GVHD developed (figure 6A, 6B). The frequency of uTh2 is also significant higher in acute GVHD and tends to be higher in chronic GVHD. The ratio of uTh1/uTh2 is significant lower in “No GVHD-external control” and tends to be lower in “No GVHD-internal control” when comparing to that of acute and chronic GVHD (figure 6C). The Pearson correlation test shows a perfect positive relationship between the frequency of uTh1 and uTh2 (r = 0.951, p<0.001; Figure 6D).

With in vitro “PMA+I” stimulation, the frequencies of sTh1 and sTh2 were still much lower in patients without GVHD (both external and internal control) and were highest in patients having acute GVHD. There was also a trend of higher sTh1/sTh2 ratio in patients having chronic GVHD (figure S4A-S4C). The Pearson correlation test also showed a positive relationship between the frequency of sTh1 and sTh2 (r = 0.875, p<0.001, figure S4D).

## Discussion

Here we demonstrate that post-transplant plasma IL-10 level and the frequency of Th1 and Th2 in CD4^+^ T cells correlate well with the clinical activity of GVHD. The plasma IL-10 level especially correlates well with the clinical activity of GVHD at all sites (gut, oral mucosa, and liver). For patients without GVHD (external control), the plasma IL-10 levels were consistently low during the “whole” post-transplant course. For patients with GVHD, the kinetic of IL-10 was highly dependent on the clinical activity of GVHD, which may occur at any post-transplant time point (as was shown in the figures). It is therefore clear that the cytokine change after transplantation is “GVHD-related” rather than “time-related”. More importantly, when GVHD responded to immunosuppressive treatment, the decrease of plasma interlukin-10 level is prompt and earlier than improvement of clinical parameters. Plasma IL-10 can therefore be served as an ideal biomarker in monitoring the clinical activity and therapeutic efficacy of GVHD. The reason why plasma IL-10 increased during active GVHD can not be answered directly in this study. It could be a compensatory phenomenon to counteract the immune reaction of GVHD as IL-10 is usually thought to be an inhibitory cytokine [25], [26]. However, IL-10 has some immunostimulatory properties as well [26]–[29]. In our study, IL-10 levels were very low in patients without GVHD (both external and internal control). Even more interestingly, the plasma IL-10 was significantly lower in “external control” (patients with no GVHD after transplant) comparing to “internal control” (inactive phase of patients having GVHD), indicates IL-10, while attenuating GVHD in some animal models [30] and ex vivo study [31], is not prerequisite to suppress the development of GVHD in the real world situation. An elegant study conducted by Miura et al had also demonstrated that markedly increased mRNA levels of IL-10 and IFN-γ could be found in patients of autologous GVHD, indicating these two cytokines may be critical mediators for the development of autologous GVHD [32]. Based on these observations, the role of IL-10 is much complex in the post-transplantation setting and may not simply be an inhibitory cytokine. The relationship between plasma IL-4 and IL-12 and GVHD is difficult to determine from this study because almost all the plasma IL-4 and IL-12 examined were below the detectable limit irrespective the clinical activity of GVHD. A more sensitive assay may be needed to clarify the role of IL-4 and IL-12 in the immune reaction and T cell polarization of GVHD.

Without in vitro “PMA+I” stimulation, the CD4^+^IFN-γ^+^ Th1 and CD4^+^IL-4^+^ Th2 cells could be easily detected in peripheral blood of patients who had GVHD, indicates the intracellular IFN-γ and IL-4 were abundant in the T helper subsets during period of GVHD. The frequency of Th1 was significant higher in both acute and chronic GVHD. The frequency of Th2 is also significant higher in acute GVHD and tends to be higher in chronic GVHD. Our data indicate both Th1 and Th2 likely contribute to the pathogenesis of GVHD. Besides, the ratio of uTh1/uTh2 tended to be lower in “No GVHD-internal control” and was significantly lower in “No GVHD-external control” comparing to that of both acute and chronic GVHD, suggests T cell polarization skewing toward a Th1 reaction in patients having acute and/or chronic GVHD. This result is somewhat conflicting to few animal studies, which demonstrated that Th2 cells have protective effects on acute GVHD [33]–[35] and may be responsible for the development of chronic GVHD [6], [7]. Nevertheless, our observation is based on the daily clinical practice and prophylactic cyclosporine-A and methotrexate were routinely administered to the transplant patients. This immunological environment is quite different to the animal models for which no cyclosporine-A/methotrexate was given and the manipulation of Th1/Th2 was tightly controlled.

Although the most clearly defined differentiation inducers for Th1 and Th2 from naïve T cells are themselves cytokines, we found the timing of changing cytokines and Th1/Th2 subsets in the blood are not earlier than the clinical onset of GVHD. Kinetics of these immunological parameters coincided with, rather than predicted, the clinical activity of GVHD. A more sensitive marker which triggers the immune reaction, i.e., the increased production of IL10 and IFN-γ, and polarization of Th1 and Th2 cells at the same time, will be needed to predict the onset of GVHD in advance. IL-21 merits further study in this regard because it potently augments the production of IL-10 and IFN-γ by various immune cells [36]–[38] and promotes the function of Th1 and Th2 [39]–[41]. Blockade of IL-21 was shown to reduce the incidence and severity of GVHD in animal studies [42]–[43]. Using the same approach to this study, we are now looking at the dynamic change of plasma IL-21 during the clinical course of GVHD. Finally, immunosuppressive therapies can have tremendous impact on the T cell subsets and cytokines. In the study of GVHD on clinical patients, it is difficult to clarify the changing immune status being due to changing status of GVHD or simply due to the effect of immunosuppressive drugs, or both. In this study, all the patients treated with identical GVHD prophylactic and therapeutic protocol. The kinetics of these immunological parameters probably reflects the collective outcome of immune reaction within the patient.

In conclusion, plasma IL-10 and the frequency of CD4^+^IFN-γ^+^ (Th1) cell and CD4^+^IL-4^+^ (Th2) cell within total CD4^+^ T cells correlate well with the activity of GVHD in the real world situation and thus can be served as good biomarkers in monitoring the clinical course and therapeutic responsiveness of GVHD. Our study also highlights the complexity of immune reaction of GVHD. Theoretically counteracting cytokines (IL-10 and IFN-γ) and T helper subsets (Th1 and Th2) increased at the same time during acute and chronic GVHD and decreased when GVHD resolved. They are all probably involved in the pathogenesis of GVHD. This complexity and highly dynamic nature of GVHD also imply the results of immunological study of GVHD should be interpreted carefully. Within a time period as short as one week, the immune parameters could be totally different on the same patient. Analysis of immune parameters at a single or only few time points after transplantation can not reflect the whole picture of post-transplant course and the result could be misleading.

## Supporting Information

Figure S1 **High plasma IL-10 level and blood-stream infection.** In our preliminary study, high plasma IL-10 levels were found in 2 patients having blood stream infection but no GVHD (Patient No. 1 and 2). Supplementary figure 1B had been presented as poster on annual meeting of European Hematology Association in 2006.
(TIF)

Figure S2 **A patient without GVHD (Patient No. 3).** The plasma levels of IL-10 and IFN-γ (figure S2A) and the frequency of uTh1 and uTh2 (figure S2B) were very low during the post-transplant course.
(TIF)

Figure S3 **Plasma IL-4 and IL-12 are very low after allotransplant.** S3A, S3B, and S3C represent the 3 patients with acute and chronic GVHD (Patient No. 2, 9, and 10 respectively). Plasma IL-4 and IL-12 levels are almost below the detectable limit and do not change with the activity of GVHD.
(TIF)

Figure S4 **Correlation between sTh1, sTh2, and GVHD.** The frequencies of sTh1 and sTh2 are significantly higher when patients had GVHD (figure S4A/S4B, student *t*-test). The sTh1/sTh2 ratio was slightly higher in patients with chronic GVHD (figure S4C). Pearson correlation test shows positive correlation between the frequency of sTh1 and sTh2 (figure S4D). Bar represents mean and error bar represents 2X standard error of the mean. (figure S4A/S4B/S4C).
(TIF)

Table S1 **Characteristics of patients enrolled in this study.**
(DOC)
