# Supplementary figures and images for: Kinetics of T Helper Subsets and Associated Cytokines Correlate Well with the Clinical Activity of Graft-Versus-Host Disease
Source: PLoS One. 2012 Sep 5;7(9):e44416. doi: 10.1371/journal.pone.0044416 (PMC3434128; doi:10.1371/journal.pone.0044416)

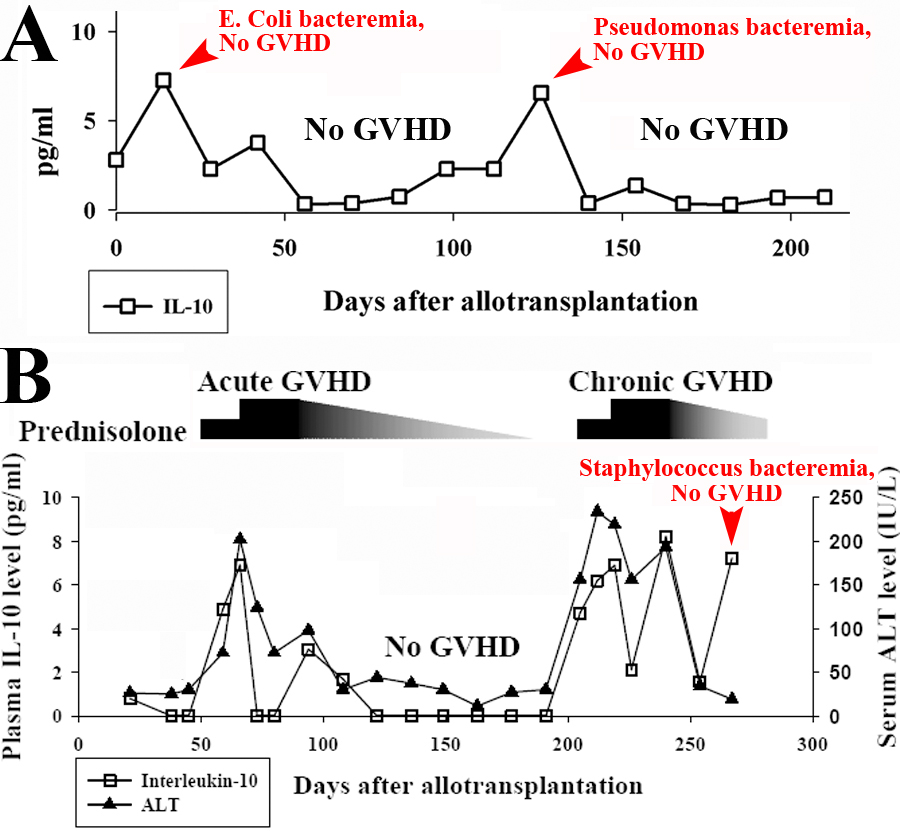

Supplement: Figure S1 — High plasma IL-10 level and blood-stream infection. In our preliminary study, high plasma IL-10 levels were found in 2 patients having blood stream infection but no GVHD (Patient No. 1 and 2). Supplementary figure 1B had been presented as poster on annual meeting of European Hematology Association in 2006. (TIF) [file pone.0044416.s001.tif]

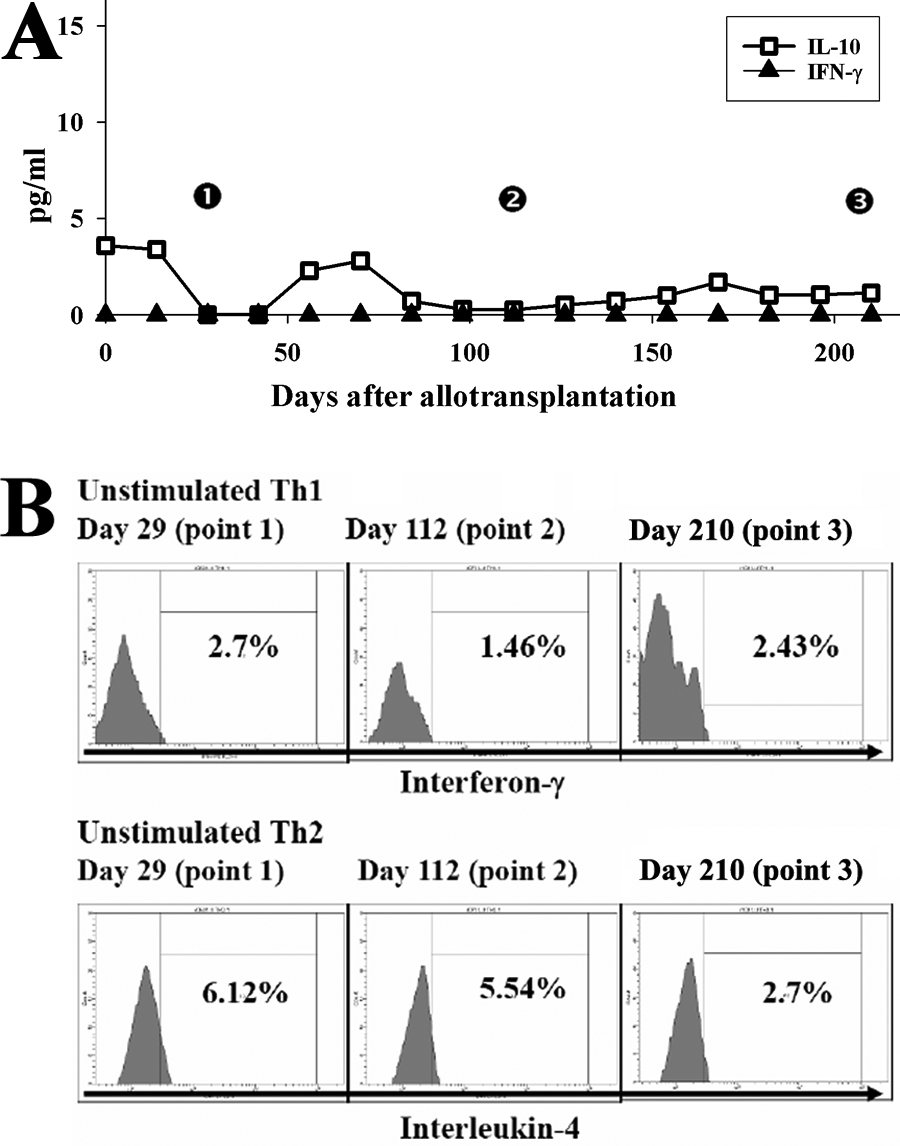

Supplement: Figure S2 — A patient without GVHD (Patient No. 3). The plasma levels of IL-10 and IFN-γ (figure S2A) and the frequency of uTh1 and uTh2 (figure S2B) were very low during the post-transplant course. (TIF) [file pone.0044416.s002.tif]

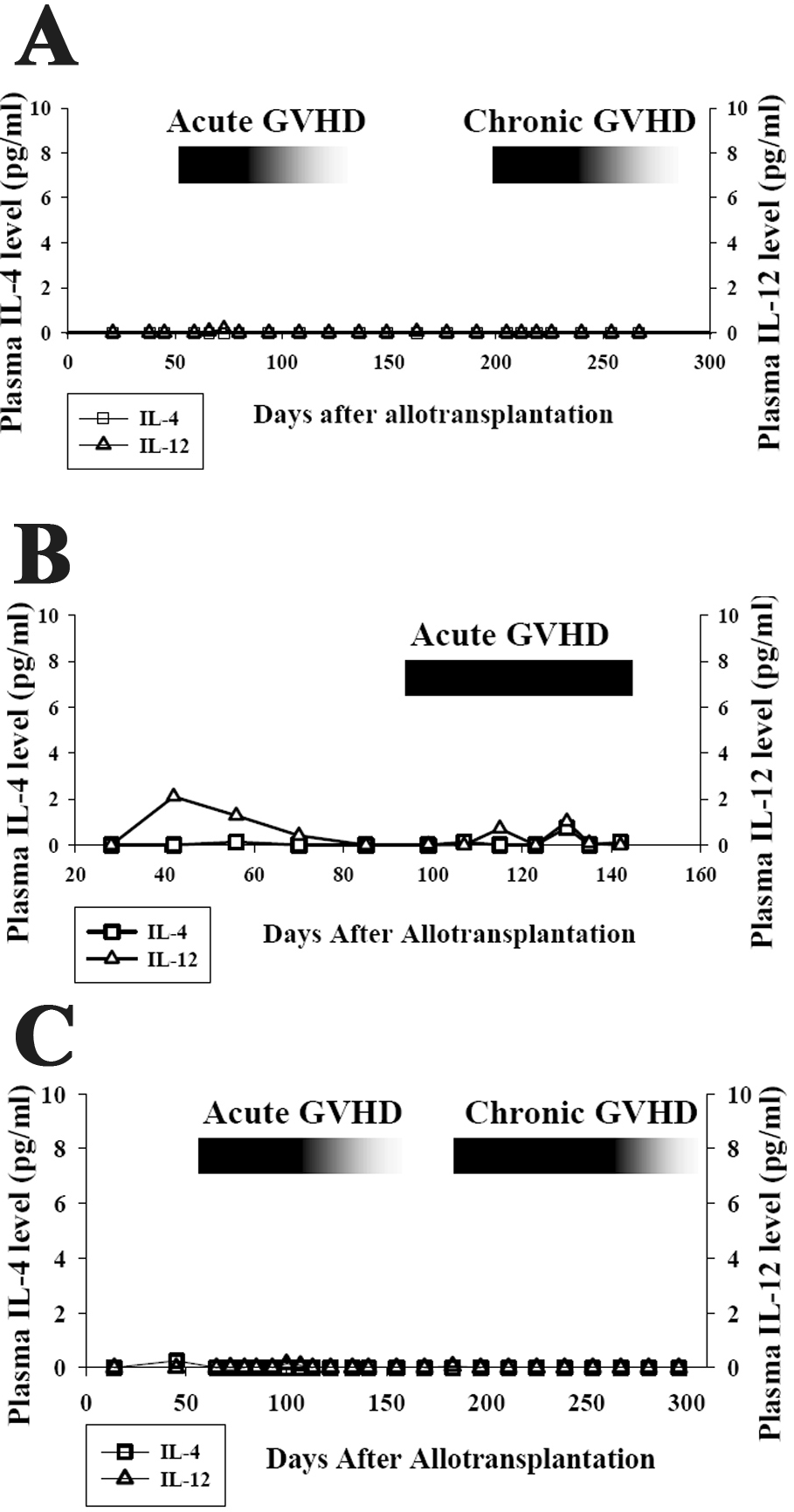

Supplement: Figure S3 — Plasma IL-4 and IL-12 are very low after allotransplant. S3A, S3B, and S3C represent the 3 patients with acute and chronic GVHD (Patient No. 2, 9, and 10 respectively). Plasma IL-4 and IL-12 levels are almost below the detectable limit and do not change with the activity of GVHD. (TIF) [file pone.0044416.s003.tif]

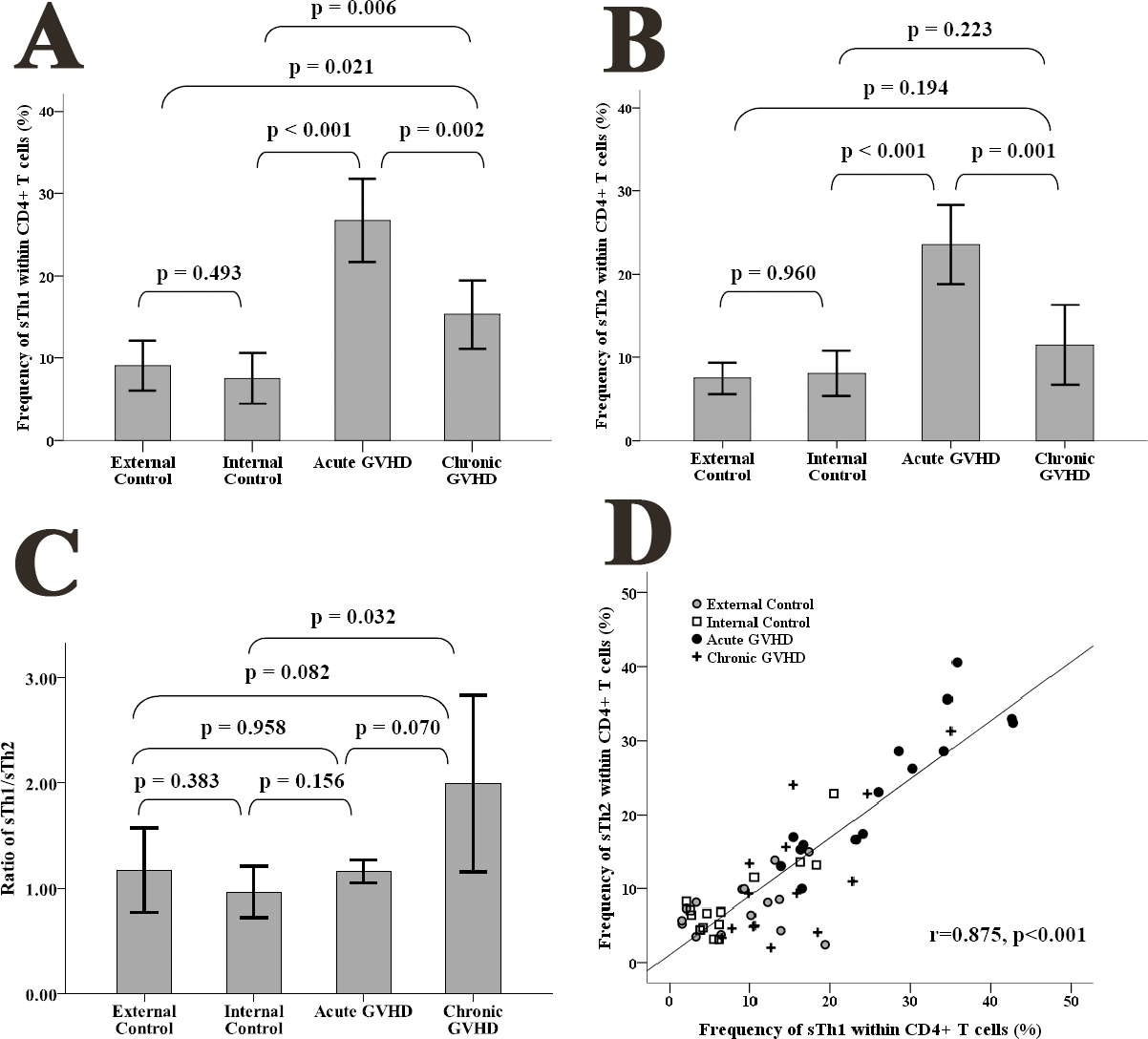

Supplement: Figure S4 — Correlation between sTh1, sTh2, and GVHD. The frequencies of sTh1 and sTh2 are significantly higher when patients had GVHD (figure S4A/S4B, student t-test). The sTh1/sTh2 ratio was slightly higher in patients with chronic GVHD (figure S4C). Pearson correlation test shows positive correlation between the frequency of sTh1 and sTh2 (figure S4D). Bar represents mean and error bar represents 2X standard error of the mean. (figure S4A/S4B/S4C). (TIF) [file pone.0044416.s004.tif]
