# Supplementary material for: Kinetics of T Helper Subsets and Associated Cytokines Correlate Well with the Clinical Activity of Graft-Versus-Host Disease
Source: PLoS One. 2012 Sep 5;7(9):e44416. doi: 10.1371/journal.pone.0044416 (PMC3434128; doi:10.1371/journal.pone.0044416)
Supplement: Table S1 — Characteristics of patients enrolled in this study. (DOC) [file pone.0044416.s005.doc]

**Supplementary Table 1: Characteristics of patients enrolled in this study**

|  | **Patient** | **Disease** | **Age/Gender** | **Conditioning**  **Regimen^a^** | **GVHD (Grade)^b^** | **Onset Day** | **Target Organs** | **Cause of Death (Time)^c^** |
| --- | --- | --- | --- | --- | --- | --- | --- | --- |
| Preliminary study on **patients with documented bacteremias** | No.1 | MM | 41/F | Mel | No (bacteremia; supplement figure 1A) **^d^** | NA | NA | Survival |
|  | No.2 | ALL | 22/M | TBI/Cy | Acute (II) 🡪 Chronic (E) (also bacteremia, supplement figure 1B)**^d^** | Day 45 (acute GVHD), Day 205 (chronic GVHD) | Skin**^e^** / oral cavity / liver**^e^** (acute GVHD), skin / liver (chronic GVHD) | Leukemia (M26) |
| **Patients without GVHD** | No.3 | AML | 38/F | BuCy2 | No (supplement figure 2A)**^d^** | NA | NA | Survival |
|  | No.4 | AML | 46/M | BuCy2 | No | NA | NA | Survival |
|  | No.5 | AML | 26/F | BuCy2 | No | NA | NA | Leukemia (M13) |
|  | No.6 | AML | 32/M | BuCy2 | No | NA | NA | Survival |
| **Patients with acute GVHD** | No.7 | ALL | 18/M | TBI/Cy | Acute (I) | Day 62 | Skin | Survival |
|  | No.8 | AML | 41/F | BuCy2 | Acute (II) | Day 45 | Skin**^e^**, oral cavity, eyes | Survival |
|  | No.9 | MDS | 32/F | BuCy2 | Acute (IV) | Day 86 | Skin**^e^**, Gut**^e^**, Liver | GVHD (M4) |
| **Patients with both acute and chronic GVHD** | No.10 | CML | 16/M | BuCy2 | Acute (III) 🡪 Chronic (E)  (Figure 2)**^d^** | Day 79 (acute GVHD); Day 197 (chronic GVHD) | Skin / oral cavity**^e^** / liver (acute GVHD), liver**^e^** / eyes /oral cavity**^e^** (chronic GVHD) | Survival |
|  | No.11 | AML | 27/M | TBI/Cy | Acute (II) 🡪 Chronic (E)  (Figure 3)**^d^** | Day 92 (acute GVHD), progressed into chronic GVHD | Skin**^e^** / liver (acute GVHD), oral cavity / eyes / liver**^e^** (chronic GVHD) | Pneumonia (M25) |
|  | No.12 | ALL | 30/M | TBI/Cy | Acute (II) 🡪 Chronic (E) | Day 33 (acute GVHD), Day 195 (chronic GVHD) | Skin (acute GVHD), oral cavity / liver**^e^** (chronic GVHD) | Survival |
|  | No.13 | AML | 32/M | BuCy2 | Acute (II) 🡪 Chronic (E) | Day 25 (acute GVHD), progressed into chronic GVHD | Gut**^e^** (acute GVHD), oral cavity / liver (chronic GVHD) | Leukemia (M21) |
| **Patients with both de novo chronic GVHD** | No.14 | ALL | 30/F | TBI/Cy | Chronic (E) | Day 134 | Eyes, skin**^e^**, oral cavity | Survival |
|  | No.15 | AML | 28/F | BuCy2 | Chronic (E)  (Figure 4A)**^d^** | Day 110 (first onset, biopsy proved on Day 182) | Liver**^e^** and lung | Survival |
|  | No.16 | AML | 43/F | BuCy2 | Chronic (E)  (Figure 4B)**^d^** | Day 175 | Liver**^e^**, eyes, skin | Survival |
|  | No.17 | AML | 42/M | BuCy2 | Chronic (L) | Day 183 | Skin**^e^** | Leukemia (M38) |
|  | No.18 | AML | 37/F | BuCy2 | Chronic (L) | Day 126 | Skin | Survival |

^a^ Conditioning regimen: Mel: Melphalan; Bu: Busulfan; Cy: Cyclophosphamide; TBI: total body irradiation

^b^ GVHD (Grade): chronic GVHD, L: limited; E: extensive

^c^ Cause of death (time): M: months after transplantation; D: days after transplantation

^d^ The patients who were showed in the figures

**^e^** Biopsy-proved GVHD
